# Engineering of Impact Ionization Characteristics in GaAs/GaAsBi Multiple Quantum Well Avalanche Photodiodes

**DOI:** 10.1021/acsphotonics.4c01343

**Published:** 2024-11-08

**Authors:** Xiaofeng Tao, Xiao Jin, Shiyuan Gao, Xin Yi, Yuchen Liu, Thomas B. O. Rockett, Nicholas. J. Bailey, Faezah Harun, Nada A. Adham, Chee H Tan, Robert D. Richards, John P. R. David

**Affiliations:** † Department of Electronic and Electrical Engineering, University of Sheffield, Sheffield S1 3JD, United Kingdom; ‡ School of Engineering & Physical Sciences, Heriot-Watt’s University, Edinburgh EH14 4AS, United Kingdom; § British Malaysian Institute, Universiti Kuala Lumpur, 53100 Kuala Lumpur, Malaysia

**Keywords:** impact ionization, avalanche multiplication, avalanche photodiodes, multiple quantum wells, GaAsBi

## Abstract

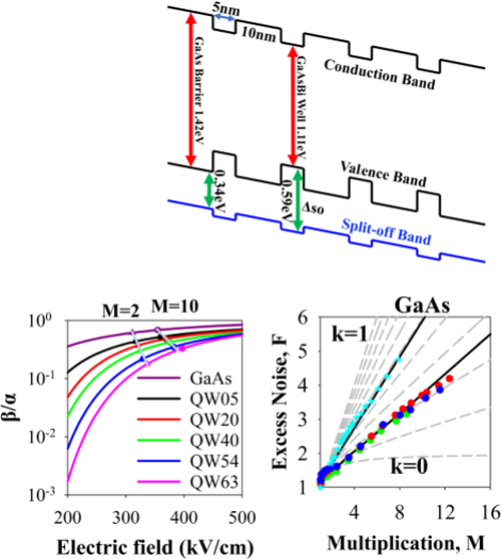

The presence of large bismuth (Bi) atoms has been shown to increase the spin–orbit splitting energy in bulk GaAsBi, reducing the hole ionization coefficient (β) and thereby reducing the excess noise seen in avalanche photodiodes. In this study, we show that even very thin layers of GaAsBi introduced as quantum wells (QWs) in a GaAs matrix exhibit a significant reduction of β while leaving the electron ionization coefficient, α, largely unchanged. The optical and avalanche multiplication properties of a series of GaAsBi/GaAs multiple quantum well (MQW) p-i-n structures with nominally 5 nm thick, 4.4% Bi GaAsBi QWs, varying from 5 to 63 periods and corresponding barrier widths of 101 to 4 nm were investigated. From photoluminescence, ω-2θ X-ray diffraction, and cross section transmission electron microscopy measurements, the material was found to be of high quality despite the strain introduced by the Bi in all except the samples with 54 and 63 QW periods. Photomultiplication measurements undertaken with different wavelengths showed that α in these MQW structures did not change appreciably with the number of QWs; however, β decreased significantly, especially at lower values, the noise factor, *F*, is reduced by 58% to 3.5 at a multiplication of 10, compared to a similar thickness bulk GaAs structure without any Bi. This result suggests that Bi-containing QWs could be introduced into the avalanching regions of APDs as a way of reducing their excess noise.

## Introduction

Semiconductor-based avalanche photodiodes (APDs) are often employed instead of normal photodiodes when there is a limited photon availability, as a means of enhancing the sensitivity of an optical system.^1^ APDs boost the signal-to-noise ratio (SNR) through a process of internal multiplication (*M*) arising from the impact ionization of optically generated carriers when the semiconductor material is subject to a high electric field. The multiplication (or gain) of the signal, however, is usually accompanied by some extra “excess” noise that arises due to the stochastic nature of the impact ionization process in semiconductors. In 1966, McIntyre defined this excess noise factor (*F*) as a function of the multiplication (*M*) as^2^
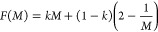
1Here, “*k*” represents the ratio of the impact ionization coefficients for holes (β) and electrons (α), denoted as *k* = β/α for the case when electrons initiate the avalanche multiplication process. This excess noise sets a limit on the maximum useful multiplication for a given device before the SNR degrades. High-sensitivity APDs require a substantial SNR, necessitating the use of avalanche materials with a very low “*k*” value for electron-initiated multiplication. Semiconductor materials like HgCdTe^3^ and InAs^4^ have effectively no hole ionization and therefore provide near-ideal multiplication with little or no excess noise; however, their narrow bandgaps mean that the devices have to be operated at cryogenic temperatures to reduce their thermally generated dark currents. The best example of a wider-bandgap semiconductor capable of low dark currents at room temperature and possessing a small *k* is silicon^5^ and AlGaAsSb.^6,7^ In an attempt to overcome the limitations of materials that have broadly similar α and β, considerable effort has gone into modifying material properties for example by using multiple quantum wells (MQWs)^8^ or “staircase” structures where band discontinuities are used to give carriers extra energy,^9^ the use of quantum dot avalanching regions,^10^ or by using nanostructuring to make one carrier type ionize more readily.^11^ These have only demonstrated limited success to date and require careful design of the material combination and/or complicated growth and fabrication for this to work. Recently, we have shown that the addition of the large Group V atom, bismuth (Bi), to GaAs had a significant effect on reducing the hole ionization coefficients while leaving the electron ionization coefficients almost unaffected.^12^ This was attributed to the effect of the band anticrossing interaction of the large Bi atom on the GaAs valence band increasing the spin–orbit splitting energy (Δso).^13^ Hole ionization in GaAs relies on holes from the heavy and light hole bands scattering into the split-off band from where they can easily gain sufficient energy to impact ionize.^14^ Any increase in Δso reduces the population of holes in the split-off band and consequently reduces β. There are a couple of problems with growing thick layers of GaAsBi. One problem with adding Bi to GaAs is that the compressive strain also increases such that the critical layer thickness^15^ can be exceeded leading to the formation of misfit dislocations and higher dark currents. Relaxed GaAsBi shows improved surface roughness compared with relaxed InGaAs;^16^ however, relaxation still negatively affects the performance of GaAsBi devices.^17^ Finding alternative ways to incorporate Bi into structures is therefore important if we are to use this idea to reduce *k* by reducing β.

In this work, we demonstrate that thick, bulk GaAsBi structures are not necessary to reduce β and even introducing thin layers of GaAsBi as quantum wells (QWs) in a GaAs matrix can help improve the performance of avalanching structures. A systematic study of the avalanche multiplication of a series of GaAsBi/GaAs MQW structures grown in a p-i-n configuration is undertaken for the first time and their ionization behaviors are investigated from photomultiplication measurements.

In this paper, we decreased the β/α ratio in GaAs by suppressing its hole impact ionization through a modification of the valence band structure. Bismuth (Bi) is one of the largest atoms that can be incorporated into GaAs. The strong difference in electronegativity between it and the arsenic (As) atoms it replaces causes Bi to act as an isovalent impurity in GaAs, strongly perturbing the valence band structure. This leads to not only a significant narrowing of the bandgap via a band anticrossing interaction^18^ but more importantly for our interests, an increase in the valence band spin–orbit splitting energy.

## Experimental Results

**Figure 1:**
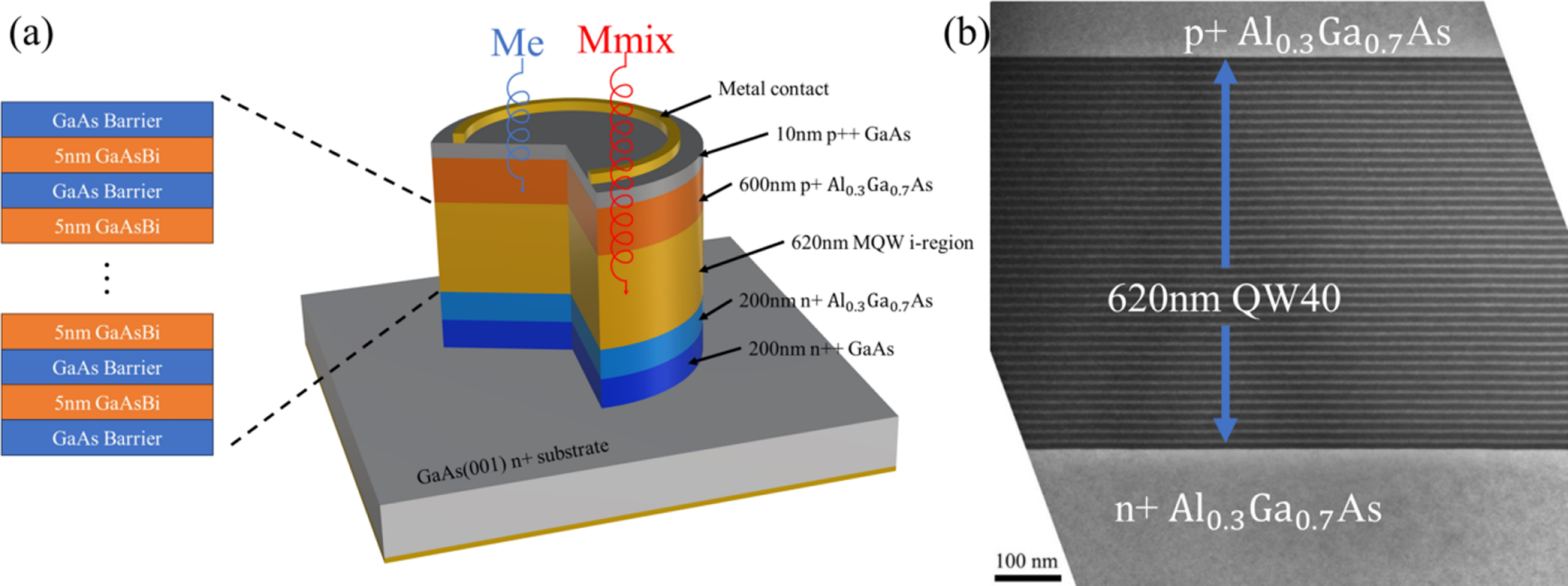
(a) Schematic cross section of the MQW p-i-n device structures used in this investigation. (b) Dark-field (002) TEM image of QW40.

A series of GaAsBi/GaAs multiple quantum well (MQW) p-i-n structures were grown on GaAs substrates with the layer structure shown in Figure 1a. The growth was paused at each well-barrier interface in an attempt to prevent the accumulation of excess bismuth on the growing surface before growing the GaAs barrier.^19^ The use of a multilayer QW structure may allow a bismuth surfactant-like layer to be present on the surface to improve the material quality,^20^ while preventing the deleterious accumulation of excess Bi on the surface that can cause roughness.^21^ The nominal GaAsBi QW thickness (*L*_w_) and Bi % (4.4%) were determined from high-resolution X-ray ω–2θ measurements (XRD), transmission electron microscopy (TEM), and photoluminescence (PL) measurements as 5 nm and 4.4% Bi, respectively.^17^ The 5 nm QW thickness and 4.4% Bi were chosen to avoid exceeding the Matthews and Blakeslee critical layer thickness^15^ while still incorporating an appreciable amount of Bi into the structures. Details of the numbers of MQW periods, which vary from 5 to 63 with corresponding barrier widths (*L*_B_), are shown in Table 1.^17,22^ For the purposes of this study, the QWs in each MQW are assumed to be identical. The calculation of the average Bi% content in the i-region (MQW_Bi%_) is shown below:
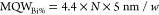
2where *N* is the number of QW’s and *w* is the total width of the i-region. On the top and bottom of the MQW region are 600 nm of p+ Al_0.3_Ga_0.7_As and 200 nm of n+ Al_0.3_Ga_0.7_As, respectively. These ensure that long-wavelength light illumination is absorbed only in the MQW region. A thin 10 nm p+ GaAs contacting layer was grown to top the structure. As demonstrated in Figure 1b, the growth resulted in uniform, evenly spaced QWs, with no obvious imperfections up to 40 periods. TEM results for other samples are shown in the Supporting Information.1

**Table 1:** Details of the MQW Structures Investigated

layer	number of periods, *N*	Barrier thickness (nm), *L*_B_	i-region thickness (nm), w	⟨MQW_Bi%_⟩
QW05	5	101	630	0.15%
QW20	20	24	605	0.70%
QW40	40	10	605	1.43%
QW54	54	6	620	2.15%
QW63	63	4	582	2.38%

**Figure 2:**
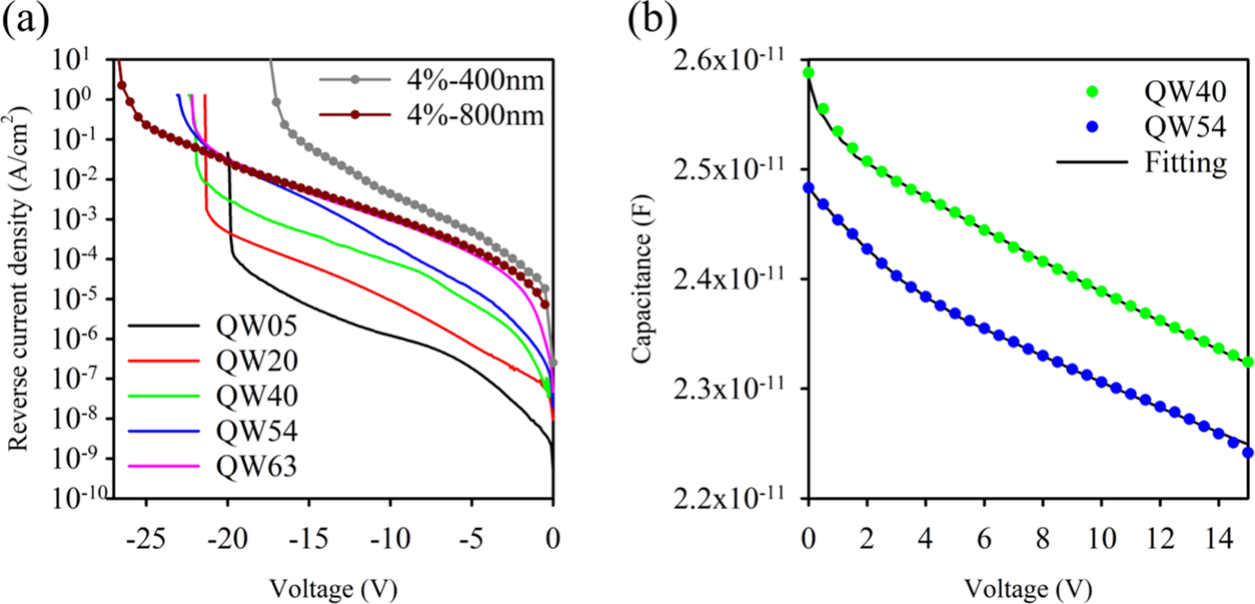
(a) Reverse dark current densities of the MQW p-i-n diodes compared with GaAs-GaAs_0.96_Bi_0.04_-GaAs p-i-n 400 and 800 nm samples. (b) Capacitance vs reverse bias of QW40 and QW54 p-i-n diodes with 200 μm radius devices. The black lines are a fit to the data.

Circular mesa diodes of several radii up to 200 μm were fabricated using standard photolithography techniques and wet etching, as shown in Figure 1a. Current–voltage (*I*–*V*) and capacitance–voltage (*C*–*V*) measurements were undertaken on the diodes and are shown in Figure 2. The dark current density in Figure 2a increases as the number of QWs increases, approaching the value we might expect in a bulk GaAsBi diode. All of the devices show the onset of avalanche breakdown at high voltages, which appears to increase as the number of QWs increases. *C*–*V* measurements in Figure 2b show that the background doping in the MQW region is low, and the decreasing capacitance with increasing reverse voltage is due to the depletion of the doped AlGaAs cladding regions. The slightly lower capacitance of QW54 is partly attributed to its slightly thicker MQW region, as shown in Table 1.

**Figure 3:**
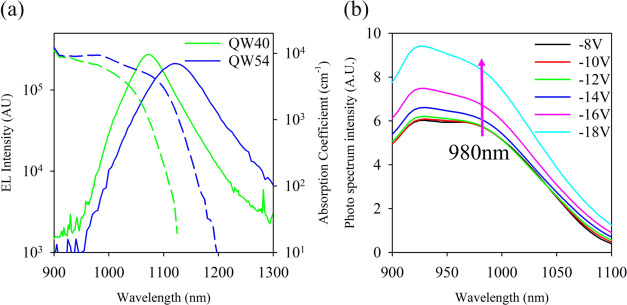
(a) Absorption coefficient (dashed lines) of QW40 and QW54, EL intensity (solid lines) of QW40 and QW54. (b) Photo spectrum of QW40 at different biases.

Avalanche multiplication measurements were undertaken by focusing light onto the optical windows of the different MQW p-i-n devices as a function of reverse bias. To extract the α and β, we ideally need to have these multiplication characteristics initiated by electrons and holes on the same device. Using 450 nm illumination, where the light is strongly absorbed in the 600 nm thick p+ Al_0.3_Ga_0.7_As layer,^23^ gave us pure electron-initiated multiplication (*M*_e_). Obtaining hole-initiated multiplication (*M*_h_) requires the complicated removal of the substrate, so we instead chose to use “mixed” carrier-initiated multiplication (*M*_mix_) where both electrons and holes are created within the MQW region using longer-wavelength light. In this situation, the absorption profile of light in the MQW region needs to be known accurately. Photocurrent measurements as a function of wavelength were obtained in these samples, and these were converted into quantum efficiency using a calibrated InGaAs photodiode. The derived absorption coefficients are shown in Figure 3a for the QW40 and QW54 samples, together with their electroluminescence (EL) spectra. The wavelength of light used must be sufficiently short that the increasing electric field in the MQW region does not affect the absorption properties due to the Franz–Keldysh^24^ or quantum-confined Stark effect^25^ but also be long enough not to be affected by absorption in the GaAs barriers. Figure 3b shows the bias dependence of the photo spectrum for QW40 and 980 nm, which was chosen as the optimum wavelength to obtain *M*_mix_ in these samples. Bias-dependent photocurrent spectra for the other samples are shown in the Supporting Information.

**Figure 4:**
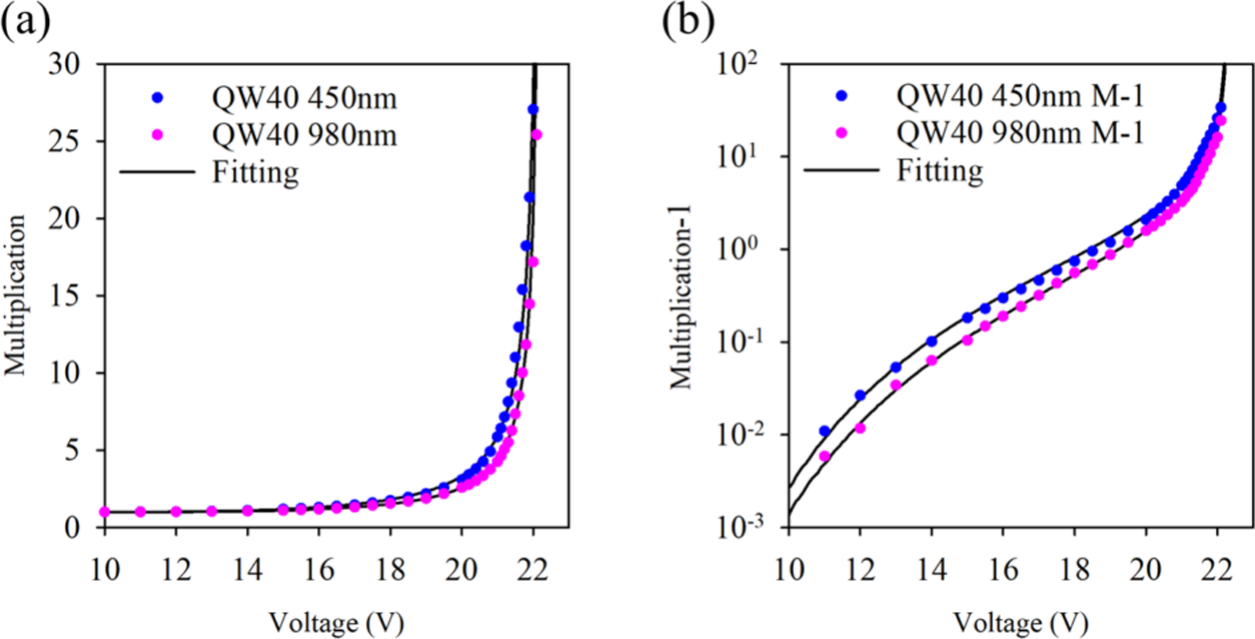
(a) *M*_e_ (blue dots) and *M*_mix_ (purple dots) of QW40 with RPL fitting (solid lines); (b) *M*_e_ – 1 (blue dots) and *M*_mix_ – 1 (purple dots) of QW40 in a log plot with RPL fitting (solid lines).

The *M*_e_ and *M*_mix_ measurements undertaken on a 200 μm diameter device of QW40 are shown in Figure 4a on a linear scale, and in Figure 4b plotted as log(*M* – 1) to accentuate the low field multiplication values. Similar measurements on the other samples are shown in the Supporting Information.

**Figure 5:**
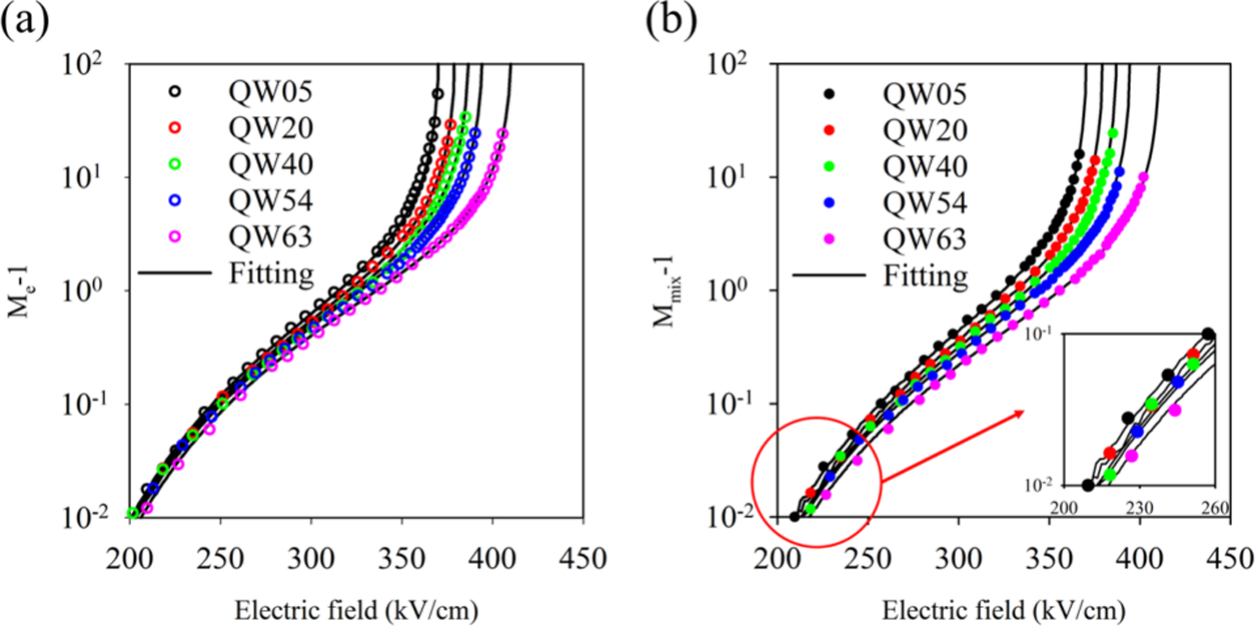
(a) *M*_e_ – 1 (dots) versus electric field of MQWs with RPL fitting (solid lines). (b) *M*_mix_ – 1 (980 nm) (dots) versus electric field of MQWs with RPL fitting (solid lines).

Similar measurements were undertaken on the other samples, and Figure 5a,b shows how *M*_e_ and *M*_mix_ vary with increasing number of wells (*N*) as a function of the reverse electric field. The data plotted as log(*M*_e_ – 1) shows that the measurable onset of the ionization process (defined here as when *M*_e_ = 1.01) occurs at a threshold electric field of around 204 kV/cm and is almost independent of the number of QWs. The threshold electric field necessary for *M*_mix_ to occur, however, varies from 209 kV/cm for QW05 and increases to 217 kV/cm for QW63.

The impact ionization coefficients were determined from these multiplication measurements using a “local” model that assumes carrier ionization at a given position within a device is a function solely of the electric field at that point (following the Chynoweth expression^26^), with no consideration of any “dead space”^27^ or history dependence of carrier energy.^28^ This dead space was found to reduce the multiplication only when the avalanching width was ≤0.1 μm^29^ and so can be ignored in these structures.

For the p-i-n devices in this study, the carriers are generated by photon absorption (*G*_a_) with an exponential decay profile dependent on the absorption coefficient (γ_λ_),^30^
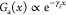
3For pure electron multiplication, all photogenerated carriers are generated prior to entering the multiplication region. In mixed multiplication, carriers are also photogenerated in the multiplier region. The observed, average multiplication is then dependent on the carrier generation function, *G*(*x*), as
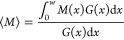
4
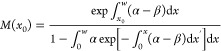
5where *M*(*x*_0_) is the multiplication due to the injection of an electron–hole pair at position *x*_0_, between the high field region 0 to *w*. In the case of p-i-n or n-i-p structures where a constant electric field can be assumed to exist between 0 and *w*, and only pure electrons or holes initiate the multiplication, this can be simplified to
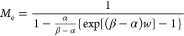
6
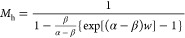
7

The α and β can be expressed as:^26^
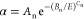
8
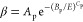
9The six coefficients, *A*_n_, *B*_n_, *C*_n_, *A*_p_, *B*_p_, and *C*_p_, are empirical coefficients that are determined from the best fit to the *M*_e_ and *M*_mix_ multiplication data. An absorption coefficient of 8000 cm^–1^ at 980 nm was assumed in this analysis. These are shown for the different structures in Table 2 and are valid for an electric field range of ∼200 to 400 kV/cm.

**Table 2:** Details of MQW Impact Ionization Coefficients

layer		*A* (105 cm^–1^)	*B* (105 V cm^–1^)	*C*
QW05	α	2.10	5.80	1.86
	β	2.00	6.45	1.9
QW20	α	2.05	5.81	1.86
	β	1.70	6.15	2.06
QW40	α	2.25	6.05	1.81
	β	1.78	6.40	2.06
QW54	α	2.20	6.07	1.81
	β	2.15	6.90	2.04
QW63	α	2.00	6.00	1.83
	β	2.10	7.00	2.1

**Figure 6:**
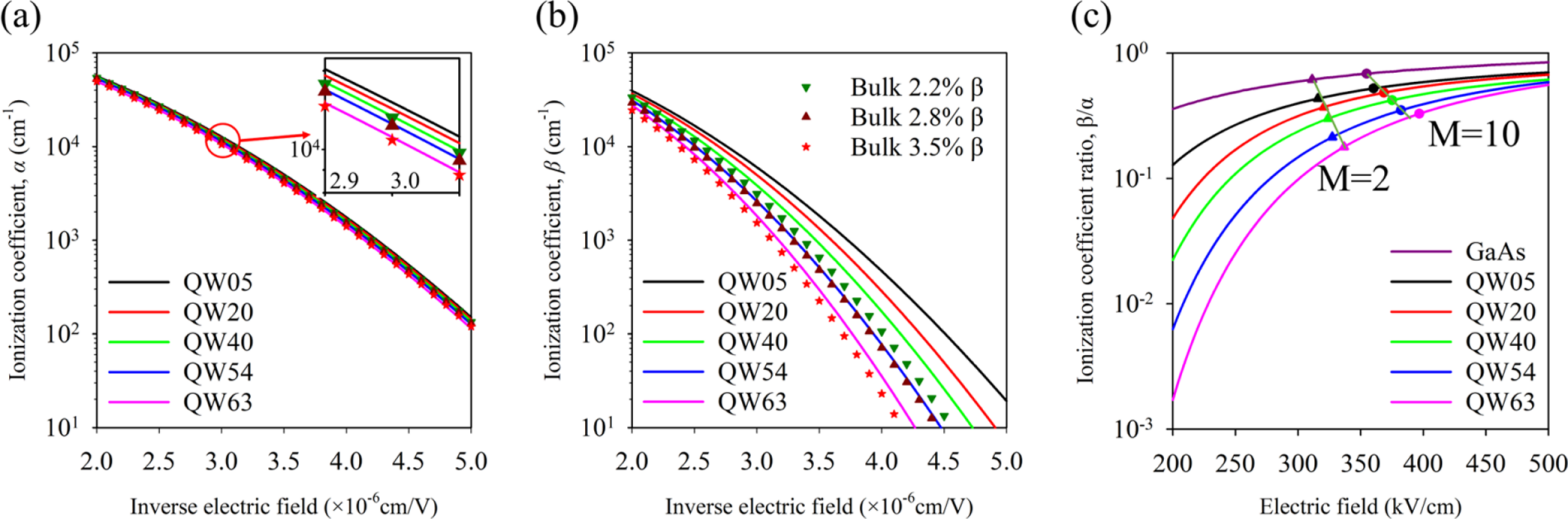
(a) α vs Inverse electric field at a range of MQWs (solid lines) and bulk GaAsBi samples,^12^ (b) β of GaAsBi vs inverse electric field for a range of MQWs and bulk GaAsBi samples,^12^ and (c) β/α ratio of MQW samples and GaAs.

Figure 6a,b shows these ionization coefficients for these MQW GaAsBi devices over a wide electric field range. The effect of the increasing *N* (effectively an increasing MQW_Bi%_) is seen more clearly in Figure 6c, where the β/α ratio (*k*) is plotted as a function of the electric field. Compared with GaAs, a significant decrease in *k* is observed (especially at lower electric fields) as *N* increases. The accuracy of these ionization coefficients is demonstrated by the random path length (RPL) simulated *M*_e_ and *M*_mix_ values for the structures, replicating the measured data almost exactly over 2 orders of magnitude as shown by the lines in Figures 4 and 5. Details of this RPL model are given in the Methods section. While the α value only decreases by about 16% between QW05 and QW63, the β value decreases by over 2 orders of magnitude at lower electric fields. Such highly dissimilar changes in ionization coefficients with increasing *N* appear to be uniquely related to the presence of GaAsBi.

**Figure 7:**
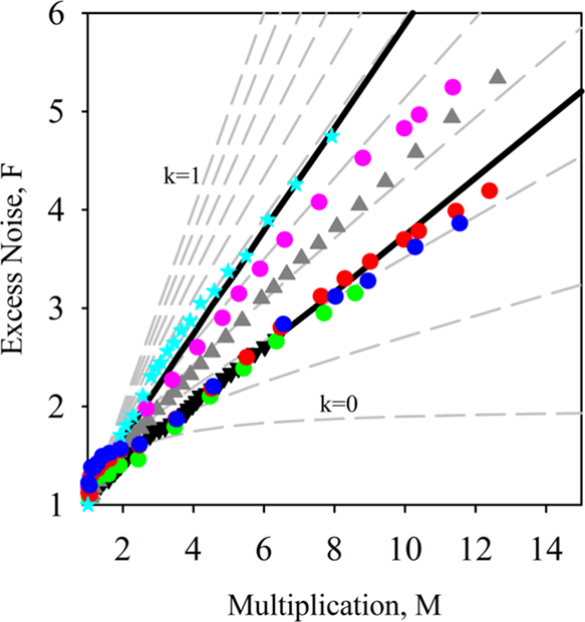
*F*_e_ results of QW40 (red, green, and blue circles) compared to that of a similar thickness GaAs bulk sample from Li et al.^32^ (cyan star). *F*_mix_ of QW40 (purple circle), *F*_e_ of bulk 450 nm GaAsBi 2.3% sample (gray triangle), and bulk 400 nm GaAsBi 4.0% bulk sample (black triangle) from Liu et al.^12^ are also shown. The higher and lower black lines are RPL simulations for the bulk GaAs and QW40, respectively, with model details as described in the text.

In order to confirm the decrease seen in *k* with an increasing number of QWs, we undertook excess noise measurements as a function of multiplication on QW40. According to eq 1, this layer should have lower excess noise than a GaAs p-i-n structure. Measurements were undertaken using the excess noise setup of Lau et al.^31^ detailed in the Methods section with 455 and 780 nm wavelength illumination, which would correspond to *M*_e_ and *M*_mix_, respectively. The results obtained are listed in Figure 7. For comparison, a GaAs p-i-n sample with an equivalent 620 nm avalanche width would have an excess noise *F* vs *M* characteristic that follows an equivalent *k* of ∼0.5^32^ as shown. The *F*_e_ measured with 455 nm on QW40 however is significantly lower, corresponding to a *k* ∼ 0.2. Using 780 nm illumination gives rise to *M*_mix_ and therefore a higher *F*_mix_ and correspondingly larger *k* as expected. Figure 7 also shows the excess noise for a 450 nm thick bulk GaAsBi 2.3% p-i-n and 400 nm thick bulk GaAsBi 4.0% p-i-n.^12^ Modeling the excess noise in structures with avalanching width <1 μm requires the ionization probability density function (PDF) to be taken into consideration^32^ as the McIntyre eq 1 is not capable of dealing with the “dead space” of the ionizing carriers. We have done this using a random path length model as described in the Methods section with the ionization coefficients for GaAs^29^ and GaAsBi from Table 2. The electron and hole threshold energies (*E*_the_ and *E*_thh_, respectively) used were *E*_the_ = 2.3 eV and *E*_thh_ = 2.1 eV for GaAs and *E*_the_ = 2.5 eV and *E*_thh_ = 3 eV for the QW40 to get the good agreement shown by the black lines in Figure 7. While these values for GaAs are broadly in keeping with previously published data,^33^ the values for QW40 are higher, suggesting that the hole ionization behavior has been disproportionally affected.

## Discussion

**Figure 8:**
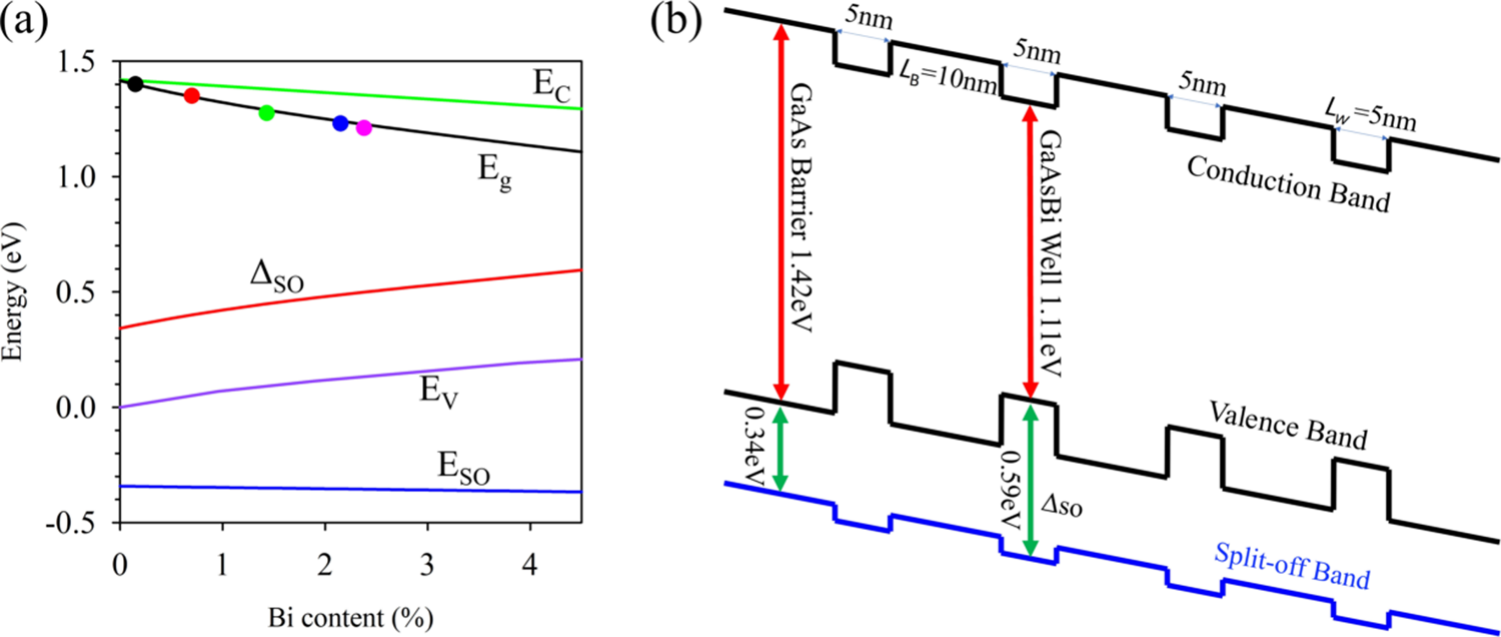
(a) Conduction band edge *E*_C_, bandgap energy *E*_g_, valence band edge *E*_V_, spin split-off band energy *E*_SO_, and spin–orbit splitting energy Δ_SO_ plotted as a function of Bi content in GaAs_1–*x*_Bi*_x_*.^13^ Colored circles represent the five MQW structures in Table 1. (b) Diagram of the conduction, valence, and split-off band energy levels of QW40.

The MQW_Bi%_ varies from 0.15 to 2.38% for the QW5 to QW63 structures respectively as shown in Table 1. The multiplication behavior of these MQW p-i-n’s is qualitatively similar to the bulk p-i-n/n-i-p structures studied by Liu et al.^12^ with the breakdown fields increasing as MQW_Bi%_ increases due to the reduction in β. The onset of *M*_e_ occurs at almost the same electric field for all of the devices (Figure 5a), but the onset of *M*_mix_ (Figure 5b) requires a slightly increasing electric field as the holes are also initiating the multiplication process. As in bulk GaAsBi structures, the α hardly reduces as *N* increases in Figure 6a compared to the β (Figure 6b), which shows a significant reduction. A comparison with the α and β of bulk GaAsBi in Figure 6a,b however shows that the MQW structure has reduced ionization coefficients, especially for β. The *F*_e_ for QW40 with MQW_Bi%_ of 1.43% in Figure 7 is also equivalent to that of a bulk GaAsBi of 4.0%, albeit with a thinner 400 nm avalanche width. To explain the mechanism, we look at the band edge energies in GaAsBi as a function of Bi%, taken from Usman et al.,^13^ as shown in Figure 8a. At low Bi%, the band gap (*E*_g_) of GaAsBi reduces because the conduction (*E*_C_) band edge reduces and valence (*E*_V_) band edges increase in energy. The split-off band energy level (*E*_SO_), however, also reduces slightly, resulting in an increase in the spin–orbit splitting energy, Δ_SO_. A consequence of this is to give rise to the band structure for the QW40 as shown in Figure 8b. The band offsets at a GaAs to GaAsBi interface are typically 40:60 for Δ_EC_:Δ_EV_.^13^ A simplistic analysis of the hole transport considering the valence band energies (shown by the black lines in Figure 8b) might expect holes to ionize more readily in the GaAsBi quantum “well”, gaining energy from Δ_EV_. However, the holes that initiate ionization will most likely have to do so from the spin split-off band^14^ as in GaAs. Looking at the energy of holes in the blue split-off band in Figure 8b, we can see that instead of gaining energy by falling into a GaAsBi “well”, the holes actually see a small barrier due to the large increase in Δ_SO_ for the GaAsBi layer. This is similar to the observation by Czajkowski et al.^34^ that ionization by electrons in a AlGaAs/GaAs quantum well was determined by the band offsets in the satellite valleys rather than Δ_EC_. This may cause β to be reduced below that which may be expected from the MQW_Bi%_ only and explain the lower measured *F*_e_ for QW40. Introducing even a few bismuth-containing QWs appears to have a beneficial advantage in reducing β, and this may be applied to other alloys as a way to reduce excess noise in APD structures.

## Conclusions

A series of GaAsBi/GaAs MQW devices with a common i-region thickness and different numbers of QWs were interrogated to investigate the effect of thin layers of GaAsBi on the impact ionization properties of GaAs. Throughout the series, the electron ionization properties α remained comparable to those of GaAs, while the hole ionization rate β was significantly reduced. This led to a reduction in overall excess noise of 3 for a device with an average Bi content of 1.43%, which is lower than what would be expected for a bulk GaAsBi layer of that Bi content. Consideration of the interfaces between the GaAsBi and GaAs band structures at energies away from the conduction and valence band edges indicates that this may be due to potential barriers experienced by highly energetic holes as they traverse the GaAsBi QWs.

## Methods

### Epitaxial Growth

The samples were grown on n+ GaAs (001) substrates by an Omicron STM-MBE system with standard Ga and Bi effusion cells and a valved cracking cell capable of producing As2 and As4. Upon loading, the samples underwent standard thermal outgas (∼350 °C) and oxide removal (∼600 °C) processes. A 200 nm n+ GaAs buffer was deposited, followed by a 200 nm n+ Al_0.3_Ga_0.7_As cladding layer. The GaAsBi/GaAs i-region was always ∼620 nm thick and contained varying numbers of GaAsBi QWs throughout the series. The QWs were evenly spaced with the GaAs barriers designed to accommodate the QWs. A p+ Al_0.3_Ga_0.7_As cladding layer was grown on top of the i-region, and this was capped with a thin p++ GaAs layer for electrical contacting.

The GaAs and GaAsBi layers were grown at ∼0.55 μm h^–1^, and the AlGaAs layers were grown at 0.79 μm h^–1^. Growth was halted for 60 s at each QW/barrier interface with the intention of preventing excess Bi buildup on the growing surface. For most of the layer, As2 was used at an atomic As:III ratio of ∼1.6; however, for the i-region, As4 was used at a stoichiometric As:III atomic ratio of 2 to enable Bi incorporation.^35^

### Device Fabrication

Circular mesa diodes with diameters ranging from 400 to 100 μm were made by conventional photolithography and wet chemical etching using equal parts of hydrobromic acid (HBr), acetic acid (CH_3_COOH), and potassium dichromate (K_2_Cr_2_O_7_). The back n-type contact was made using InGe/Au and the top p-type annular contacts with optical windows were made using Au/Zn/Au.

### Multiplication and Excess Noise Measurements

The multiplication is obtained by focusing laser light onto the optical window of a device and increasing the reverse bias voltage. Using a lock-in amplifier and modulated laser light enables any dark currents to be ignored, increasing the sensitivity of the measurement. The excess noise measurements were performed at a center frequency of 10 MHz using the measurement system described by Lau et al.^31^ A phase-sensitive technique was used to remove any contributions from dark currents, enabling excess noise to be measured at high values of multiplication. A baseline correction to the increasing photocurrent has to be applied to account for changes to the carrier collection efficiency. This baseline correction is essential because the accurate calculation of *F* is highly sensitive to small changes in the calculated multiplication.

### RPL Modeling of Excess Noise

This model employs a randomly generated ionization path length to determine where an ionization event will occur. Secondary carriers generated at this point undergo a similar process until all carriers exit the device. The ionization probability distribution functions (PDFs) of the electron and hole ionization path lengths are used in this simulation. This model can include “nonlocal” ionization by displacing the PDF by the “dead space” distance. This dead space is the minimum distance that an electron or a hole must travel to attain sufficient energy for impact ionization, calculated from the threshold energy of carriers traveling in an electric field. Further details of this model are given in Ong et al.^33^

## Data Availability

The data that support the findings of this study are available from the corresponding authors upon reasonable request.

## Supporting Information Available

Transmission electron microscope (TEM) results on QW20 and QW54; bias-dependent photocurrent spectra for QW5, QW20, QW54, and QW63; and avalanche multiplication (*M*) and *M* – 1 results on QW5, QW20, QW54, and QW63 at different wavelengths (PDF)
