# Supplementary material for: Engineering of Impact Ionization Characteristics in GaAs/GaAsBi Multiple Quantum Well Avalanche Photodiodes
Source: ACS Photonics. 2024 Nov 8;11(11):4846–53. doi: 10.1021/acsphotonics.4c01343 (PMC11583317; doi:10.1021/acsphotonics.4c01343)
Supplement: Supplementary file 1 — ph4c01343_si_001.pdf [file ph4c01343_si_001.pdf]

Supporting Information

# Engineering of impact ionization characteristics in GaAs/GaAsBi multiple quantum well avalanche photodiodes

*Xiaofeng Tao[1], Xiao Jin[1,*], Shiyuan Gao[1], Xin Yi[1,2], Yuchen Liu[1], Thomas B. O. Rockett[1], Nicholas. J. Bailey[1], Faezah Harun[1], Nada A. Adham[1], Chee H. Tan[1], Robert D. Richards[1], and John P R David[1,*]*

[1]*Department of Electronic and Electrical Engineering, University of Sheffield, Sheffield, S1 3JD, United Kingdom*

[2]*School of Engineering & Physical Sciences, Heriot-Watt's University, Edinburgh, EH14 4AS, United Kingdom*

[*]*Corresponding authors xjin4@outlook.com & j.p.david@sheffield.ac.uk*

## 1. TEM

TEM shows that QW20 below (Fig, S1a) has no defects or dislocations. When the barrier width decreases to 6 nm and the number of periods increases to 54 (Fig. S1b), dislocations start at the interface between the MQW and the cladding layers.

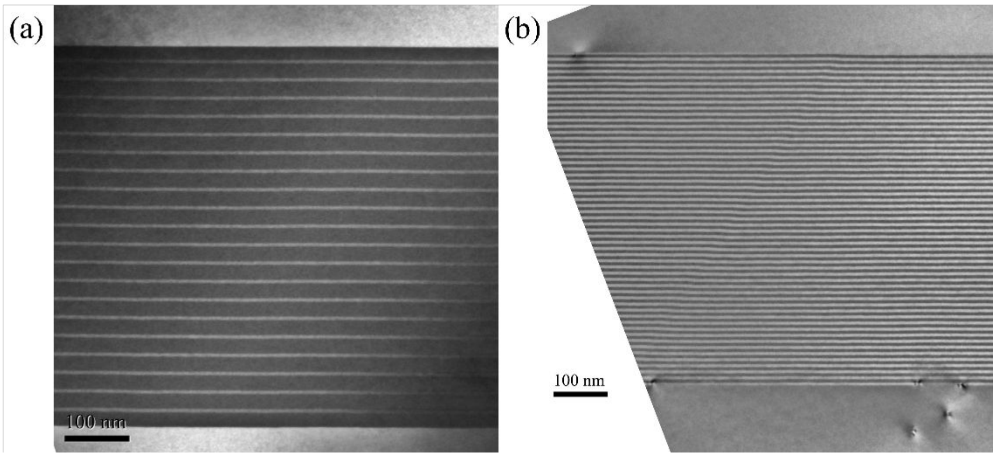


Figure S1.1 (a) TEM of QW20; (b) TEM of QW54

## 2. PL and XRD: MQW vs Bulk comparison

The PL and XRD of QW40 and QW63 are compared to those of 400 nm and 800 nm bulk 4 % GaAsBi p-i-n diodes.

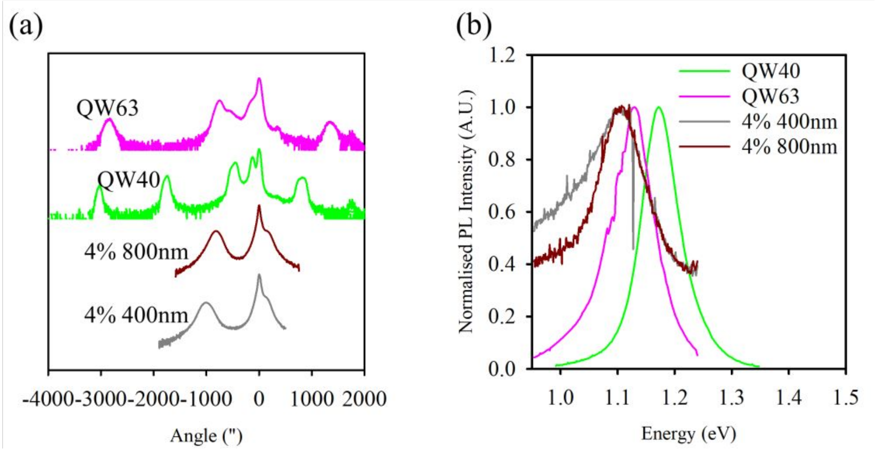


Figure S2.1(a) XRD of QW40 and QW63 compared with 4%GaAsBi bulk samples
(b) PL of QW40 and QW63 compared with 4% bulk samples renormalise the bulk PL from 0 to 1.0.

## 3. Current -Voltage characteristics: MQW vs Bulk comparison

The forward and reverse I-V characteristics of QW40 and QW63 are compared to those of 400 nm and 800 nm bulk 4 % GaAsBi p-i-n diodes

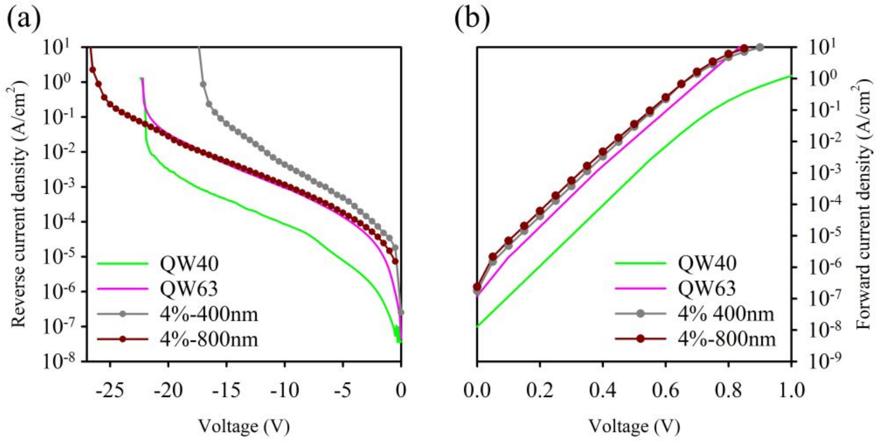


Figure S3.1 (a)Reverse current density of QW40, QW63 compared with 4.0% bulk samples; (b)Forward current density of QW40, QW63 compared with 4.0% bulk samples

## 4. Bias dependent photocurrent spectra

Figs S4.1 (a)-(d) show the photocurrent spectra as a function of reverse bias for QW05, QW20, QW54 and QW63. In all cases, illumination with 980 nm light would give rise to mixed carrier multiplication.

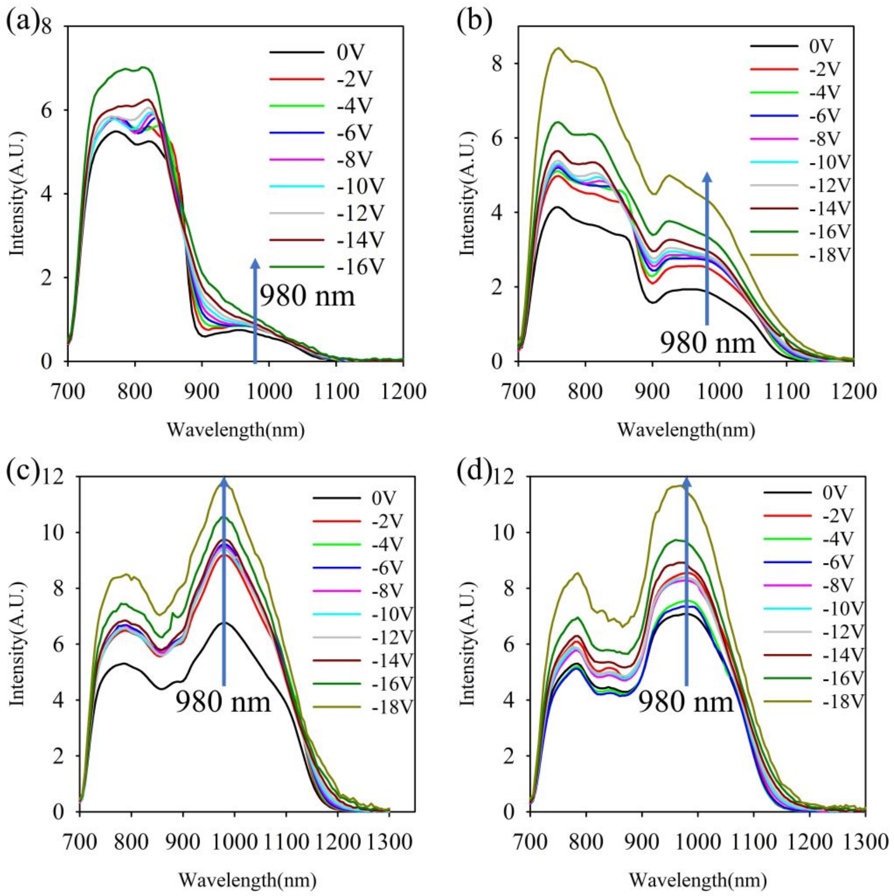


Figure S4.1 (a) Photo spectrum of QW05; (b) Photo spectrum of QW20; (c) Photo spectrum of QW54; (d) Photo spectrum of QW63 at different biases.

Fig S4.2 shows the absorption coefficient of MQWs for QW05, QW20, QW40, QW54, QW63 when they are fully depleted. [Why -2, -3, -5 V? Just say when they are fully depleted?]

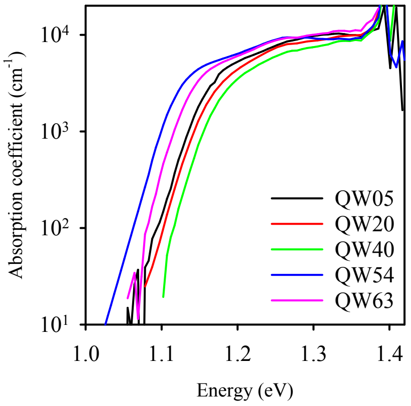


Figure S4.2 Absorption of MQWs

# 5. Multiplication characteristics

The pure electron ($M_e$) and mixed electron and hole ($M_{mix}$) carrier multiplication for QW05, QW20, QW54 and QW63 are shown in Fig. S5.1, Fig. S5.2, Fig. S5.3 and Fig. S5.4

respectively.

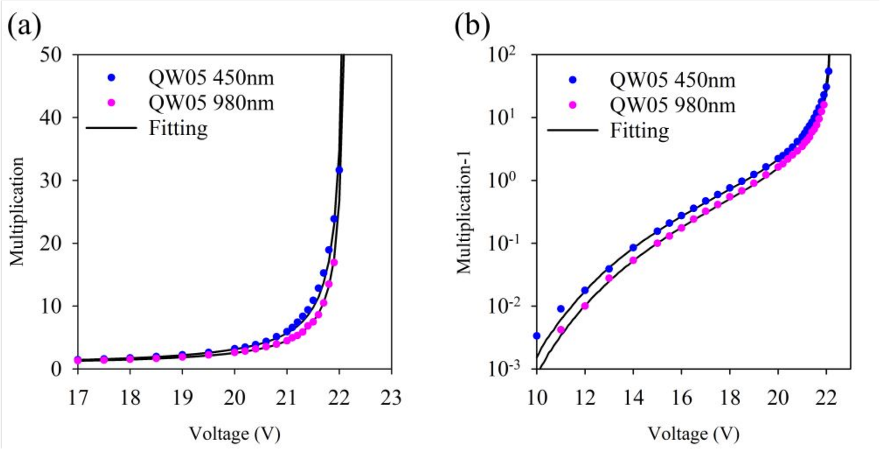


Figure S5.1 (a) $M_e$ (blue dots) and $M_{mix}$ (purple dots) of QW40 with RPL fitting (solid lines); (b) $M_e - 1$ (blue dots) and $M_{mix} - 1$ (purple dots) of QW40 in log plot with RPL fitting (solid lines).

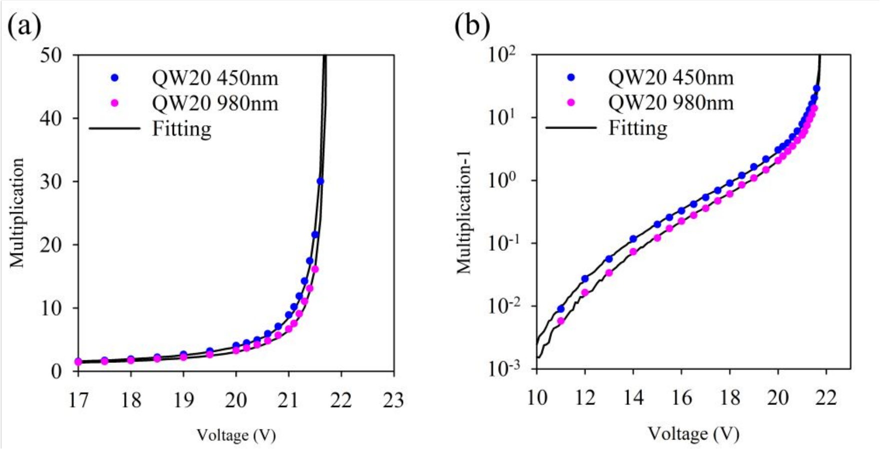


Figure S5.2 (a) $M_e$ (blue dots) and $M_{mix}$ (purple dots) of QW20 with RPL fitting (solid lines); (b) $M_e - 1$ (blue dots) and $M_{mix} - 1$ (purple dots) of QW20 in log plot with RPL fitting (solid lines).

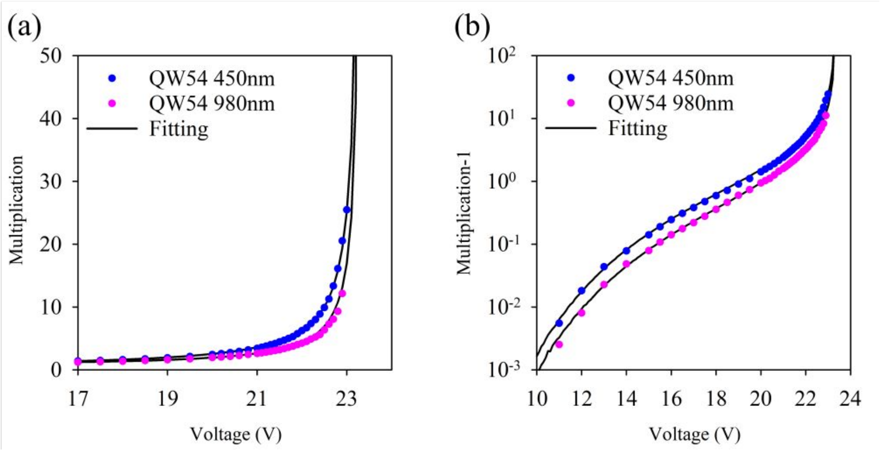


Figure S5.3 (a) $M_e$ (blue dots) and $M_{mix}$ (purple dots) of QW54 with RPL fitting (solid lines); (b) $M_e - 1$ (blue dots) and $M_{mix} - 1$ (purple dots) of QW54 in log plot with RPL fitting (solid lines).

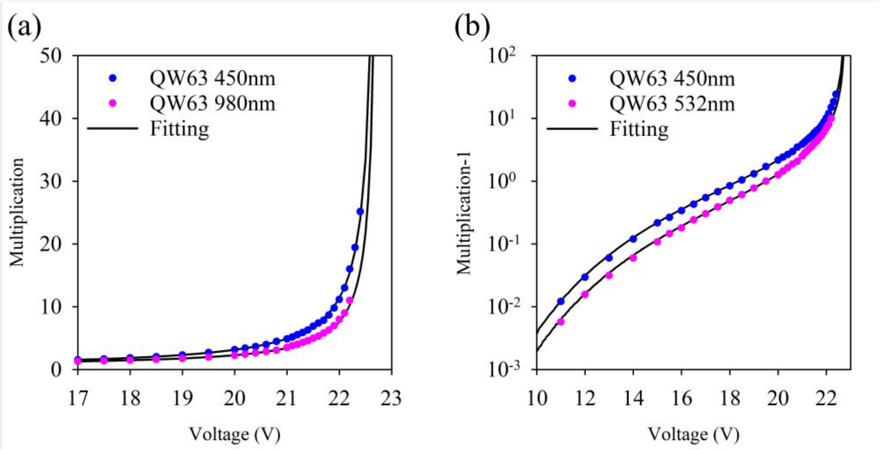


Figure S5.4 (a) $M_e$ (blue dots) and $M_{mix}$ (purple dots) of QW63 with RPL fitting (solid lines); (b) $M_e - 1$ (blue dots) and $M_{mix} - 1$ (purple dots) of QW63 in log plot with RPL fitting (solid lines).
